# Replication in Cells of Hematopoietic Origin Is Necessary for Dengue Virus Dissemination

**DOI:** 10.1371/journal.ppat.1002465

**Published:** 2012-01-05

**Authors:** Alissa M. Pham, Ryan A. Langlois, Benjamin R. tenOever

**Affiliations:** Department of Microbiology, Mount Sinai School of Medicine, New York, New York, United States of America; Purdue University, United States of America

## Abstract

Dengue virus (DENV) is a mosquito-borne pathogen for which no vaccine or specific therapeutic is available. Although it is well established that dendritic cells and macrophages are primary sites of DENV replication, it remains unclear whether non-hematopoietic cellular compartments serve as virus reservoirs. Here, we exploited hematopoietic-specific microRNA-142 (miR-142) to control virus tropism by inserting tandem target sites into the virus to restrict replication exclusively in this cell population. *In vivo* use of this virus restricted infection of CD11b^+^, CD11c^+^, and CD45^+^ cells, resulting in a loss of virus spread, regardless of the route of administration. Furthermore, sequencing of the targeted virus population that persisted at low levels, demonstrated total excision of the inserted miR-142 target sites. The complete conversion of the virus population under these selective conditions suggests that these immune cells are the predominant sources of virus amplification. Taken together, this work highlights the importance of hematopoietic cells for DENV replication and showcases an invaluable tool for the study of virus pathogenesis.

## Author Summary

Dengue virus (DENV) is becoming a global threat as anthropogenic factors are increasing the prevalence of vector species capable of transmitting the pathogen. There are currently no vaccines or therapeutics against DENV, and the study of virus pathogenesis and dissemination has been largely limited to artificial mouse models. As DENV is capable of infecting many cell types including dendritic cells (DCs), macrophages, and fibroblasts, it remains unclear which cells permit DENV replication *in vivo* and are responsible for virus spread. To this end, we inserted microRNA (miRNA) target sites into the virus genome to render it incapable of replicating in DCs and macrophages, while having no direct effect on replication in other cell types. The purpose of this study was to exclude virus growth in a defined cellular population and assess the effects of this restriction on viral dissemination and replication *in vivo*. With this technology, we demonstrate that restricting replication in hematopoietic cells results in a complete loss of miRNA-targeted virus, indicating that these cells are the predominant reservoirs for virus replication.

## Introduction

Dengue virus (DENV) is a mosquito-borne flavivirus and the etiological agent of dengue fever and the more severe clinical presentations known as dengue hemorrhagic fever (DHF) and dengue shock syndrome (DSS) [1]. The virus is endemic in tropical and subtropical regions around the world, and poses a major global health concern [2]. The four serotypes of DENV (DENV-1, -2, -3, and -4) are transmitted by *Aedes* mosquitoes which, given their global distribution, renders at least 2.5 billion people at risk of contracting the virus, resulting in as many as 10 million annual infections worldwide [2], [3]. Like other flaviviruses, the DENV genome is translated into a single polyprotein precursor that is co-translationally processed by cell- and virus-specific proteases [4]. Virus infection results in the generation of three structural proteins, C, prM and E, and seven non-structural (NS) proteins, NS1, NS2a, NS2b, NS3, NS4a, NS4b, and NS5 [1], [4]. Primary infection of DENV results in mild febrile illness and confers only partial protection from the remaining serotypes [5], [6]. Consequently, a heterotypic secondary infection with a different serotype can lead to more severe or fatal clinical manifestations such as DHF/DSS [7]. This pathogenesis feature is referred to as antibody-dependent enhancement, a phenomenon mediated by Fc receptors found on cells such as macrophages, neutrophils, and DCs [5], [6], [8]. Non-neutralizing antibodies against DENV during a heterotypic secondary infection allows for infectious immune complexes to enter Fc-receptor expressing cells and initiate replication, thereby enhancing virus growth within these cells [6].

DENV replication originates at the site of mosquito inoculation in resident cutaneous Langerhans dendritic cells (DCs), whose migration through the lymphatic system results in the induction of cytokines and the chemokine-mediated recruitment of immune cells [9], [10]. These include monocytes and macrophages, which are also known to be primary targets of DENV infection [11]. Although it is understood that these cells of hematopoietic lineage are major sites of DENV replication, it remains uncertain whether other cells of non-hematopoietic origin are permissive to virus replication during natural infection. *In vitro* studies describe a variety of cell lines of different type and host lineage (human, murine, monkey, hamster, mosquito) that are permissive to DENV replication [12]. Furthermore, the virus engages a number of different receptors including heparan sulfate, DC-specific intercellular adhesion molecule 3-grabbing nonintegrin, CD14, and heat shock proteins 70 and 90 [13]–[17]. This broad range of receptors has led some to propose endothelial cells may be targets of DENV replication during natural infection [18]–[20]. However, the interpretation of these studies has been questioned, as the presence of viral antigen in endothelial cells could be a result of virus entry rather than active replication. A counter argument in support of viral entry, in the absence of replication, is supported by results that found undetectable levels of viral RNA in endothelial cells in response to infection [21]. In this study, we sought to determine the effects of excluding DENV infection from hematopoietic cells through exploitation of the host microRNA (miRNA) machinery, and determine whether DENV replication could still be harbored in the absence of its primary cell targets.

miRNAs are small (18–22 nucleotides (nts)), trans-acting RNAs that modulate post-transcriptional silencing (PTS) of target genes by binding to miRNA response elements present in the open reading frame and/or 3′ untranslated regions (UTRs) of target transcripts [22], [23]. This activity is mediated by members of the Argonaute family, most notably, Argonaute 2 (Ago2), the catalytic member of the RNA-induced silencing complex (RISC). The Ago2-containing, miRNA-loaded RISC binds to target mRNA via the “seed” region of the miRNA (bases 2–7), or through extensive centered complementarity, resulting in translational repression or RNA degradation [24]–[26]. In contrast to miRNAs, short-interfering RNAs (siRNAs) bind with perfect complementarity and disrupt target expression levels more effectively through Ago2-mediated cleavage of the messenger RNA transcript [22], [27]–[30]. It therefore follows that incorporation of fully complementary miRNA target sites into a virus can effectively convert an endogenous miRNA into a virus-specific “siRNA”. This concept has been successfully demonstrated for a number of viruses, including influenza A virus, poliovirus, vesicular stomatitis virus, and measles virus [31]–[39]. In addition, DENV itself has been rendered susceptible to a miRNA, but only in the context of a replication-incompetent replicon or as a chimera with tick-borne encephalitis virus structural proteins [37], [40].

In an effort to exclude DENV selectively in immune cells, we incorporated perfect hematopoietic-specific miRNA target sites into the 3′ UTR of the virus to serve as a tool to study DENV replication in non-hematopoietic cells *in vivo*. We demonstrate that miR-142 targets can confer cell-specific attenuation in both *in vitro* and *in vivo* assays, and that hematopoietic cell types, such as monocytes, DCs, and macrophages, are required for DENV growth and dissemination during natural infection.

## Results/Discussion

**Figure 1:**
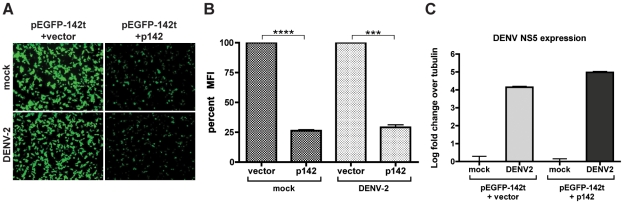
DENV-2 does not block miRNA function. (A) Fluorescence microscopy of human fibroblasts cotransfected with a plasmid expressing GFP targeted by miR-142 (pEGFP-142t) and either a construct expressing miR-142 (p142) or an empty vector control (vector). Cells were mock treated or infected with DENV-2 at an MOI of 1 48 hrs prior to analysis. (B) Fluorescence-activated cell sorting of samples treated as in (A). (C) Quantitative RT-PCR on samples described in (A). Data depicted as NS5 over tubulin levels. (Statistical significance: ****, P<0.0001, ***, P<0.001).

Before designing a method of exploiting miRNA machinery to suppress DENV replication, we sought to confirm DENV infection does not block miRNA biogenesis or function. To assess effective knockdown of a miRNA target, human fibroblasts expressing a green fluorescent protein (GFP) targeted by miR-142 (pEGFP-142t) were transfected with either vector or a plasmid expressing miR-142 (p142), a miRNA normally absent in these cells. In uninfected cells, expression of miR-142 resulted in a dramatic reduction of visible GFP expression (Figure 1A), equating to a 75% loss of fluorescence (Figure 1B). Furthermore, despite a multiplicity of infection (MOI) that would ensure high levels of virus replication, verified by quantitative RT-PCR (qRT-PCR) (Figure 1C), the presence of DENV-2 did not alter the efficiency of miR-142-mediated PTS. In accordance with the lack of DENV-2-mediated disruption of miR-142-targeting, northern blots for the expression of other exogenous (miR-124) and endogenous (miR-93) miRNAs also demonstrated no discernable differences during virus replication (Figure S1). Taken together, these results suggest DENV-2 does not interfere with miRNA-mediated PTS or miRNA biogenesis, and that cellular miRNA machinery may be exploited for DENV targeting.

**Figure 2:**
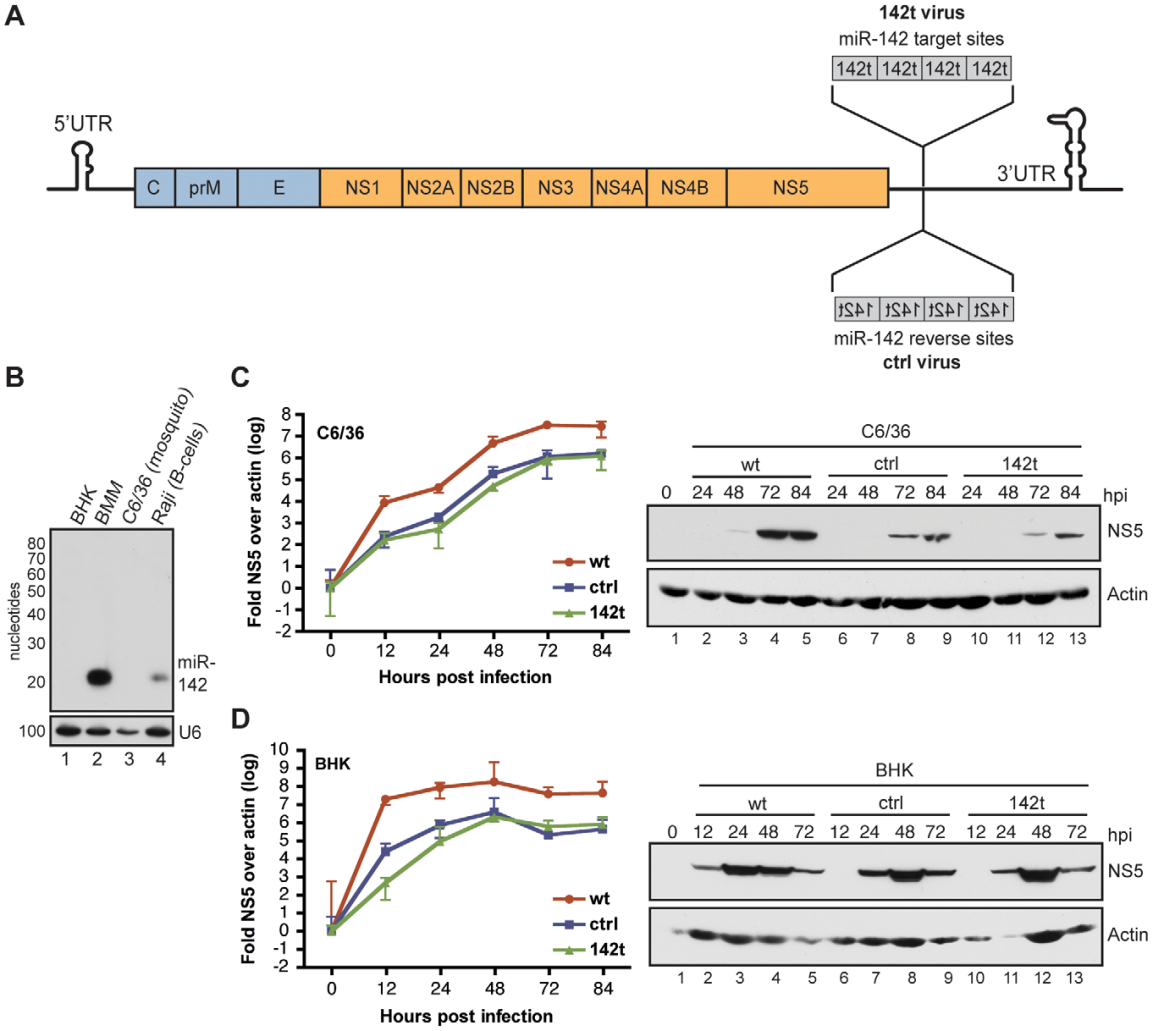
Generation and characterization of miR-142-targeted DENV-2. (A) Cloning strategy for the insertion of miR-142 target sites into the 3′untranslated region (UTR) of a T7-driven DENV-2 cDNA clone. A 157 nucleotide (nt) insert containing four tandem target sites were cloned into the variable region of DENV-2 (142t virus). A control (ctrl) virus containing four reverse sites is also depicted. (B) Northern blot for miR-142 expression in *Aedes albopictus* mosquito (C6/36) cells, mammalian baby hamster kidney cells (BHKs), murine bone-marrow-derived macrophages (BMMs), and a human B cell line (Raji). (C) Quantitative RT-PCR and western blot analysis on C6/36 cells infected with wt, ctrl, and 142t viruses at the indicated time points. Wild type (wt) virus refers to a clone encompassing no modifications. (D) Same as described in (C) for BHK cells.

During natural infection, DENV displays tropism for cells of hematopoietic lineage, specifically, monocytes, macrophages and DCs [9], [11], [36], [41], [42]. As miR-142 is one of the most abundant hematopoietic-specific miRNAs [43], [44], it served as an ideal candidate for mediating cell-specific attenuation. To generate a DENV-2 strain that demonstrated miR-142-susceptibility, we incorporated four target sites into the 3′ UTR of the virus (Figure 2A). Given the highly structured secondary conformations important for both transcription and translation in the 3′ UTR [45], [46], we incorporated the target sites downstream of the NS5 coding frame, in the variable region believed to be more amenable to nt insertions [46] (Figure S2). Following construction of both a miR-142-targeted (142t) virus and a parental control (ctrl) virus, encoding reverse target sites, we next sought to determine their replicative properties in cells lacking miR-142. To this end, we performed multi-cycle growth curves in mosquito *Aedes Albopictus* larvae cells (C6/36) and baby hamster kidney (BHK) cells, neither of which express miR-142 (Figure 2B). By qRT-PCR and western blot, both ctrl and 142t strains grew to similar levels in C6/36 cells (Figure 2C) reaching equal growth at 84 hrs post infection (hpi), but demonstrating an approximate one-log reduction as compared to wild type, unmodified virus (wt). This data was further validated by plaque assay, demonstrating peak titers of ∼1×10^3^ PFU/mL for both ctrl and 142t viruses as compared to ∼1×10^4^ PFU/mL for wt virus (Figure S3). Furthermore, experiments in BHK cells, another cell devoid of miR-142, demonstrated peak virus loads at 48 hpi, a time at which cytopathic effects reduced overall titers and NS5 expression (Figure 2D). Consistent with the data from C6/36 cells, wt virus exceeded both ctrl and 142t strains, suggesting that the 157 nt insertion lowered the overall fitness of the virus, independent of sequence.

**Figure 3:**
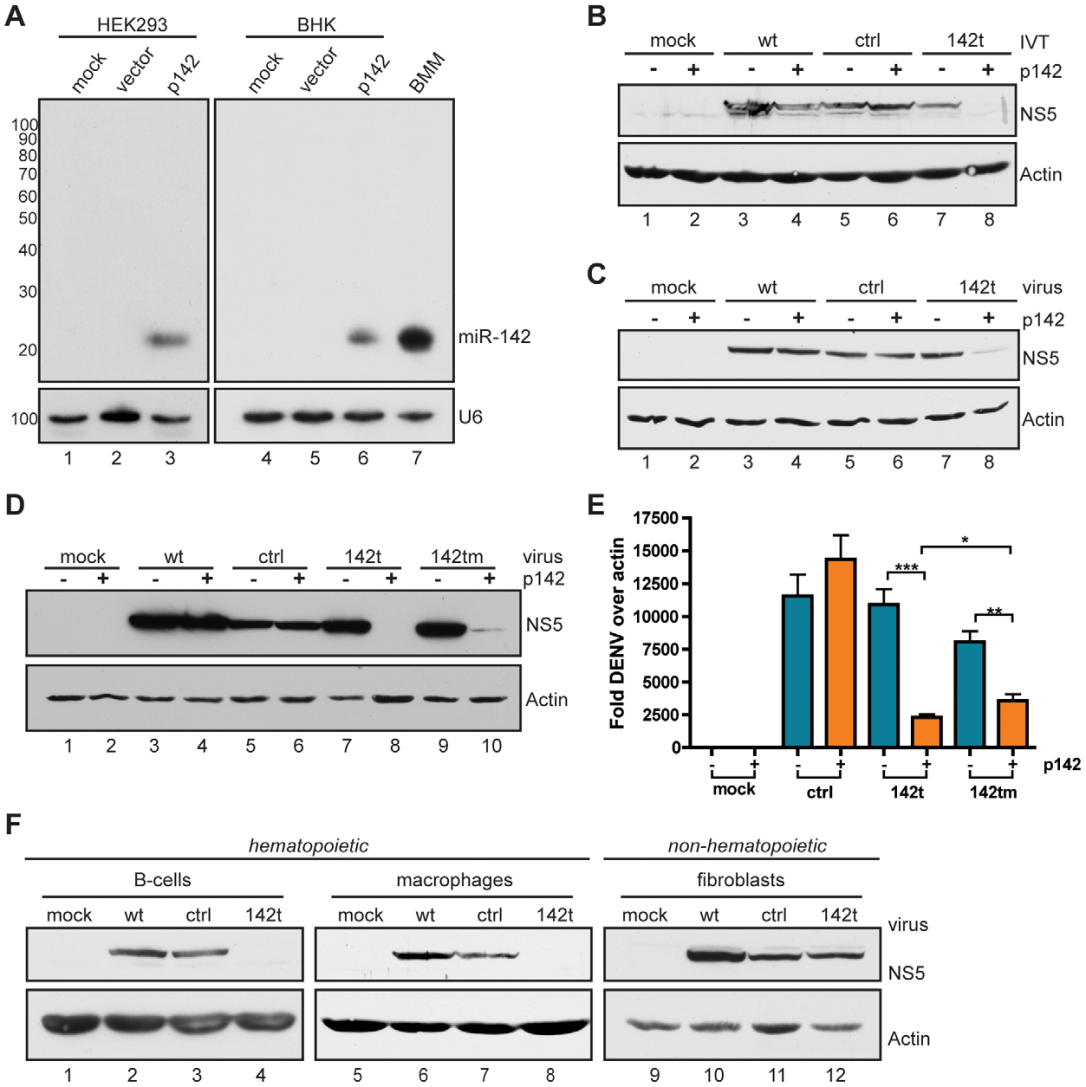
*In vitro* knockdown of miR-142-targeted DENV-2 during exogenous and endogenous miR-142 expression. (A) Northern blot confirming exogenous expression of miR-142 by plasmid transfection. BHKs and HEK293s were transfected with vector or p142 and analyzed 24 hrs post transfection (hpi). Bone marrow-derived macrophages (BMMs) were included as a positive control for miR-142 expression. (B) Western blot for NS5 and actin expression in BHKs transfected with vector (−) or p142 (+) and treated with wt, ctrl, or 142t *in vitro* transcription (IVT) products. (C) BHKs treated as in (B) were infected with wt, ctrl, or 142t viruses and analyzed as in (B). (D) Western blot for NS5 and actin expression in BHKs treated as in (B) with wt, ctrl, 142t, or 142tm IVT products. (E) Quantitative RT-PCR on DENV-2 transcript in BHKs treated as in (B) with ctrl, 142t, or 142tm IVT products. (F) Western blot for NS5 and actin expression in hematopoietic cells (Raji B cells and BMMs) and non-hematopoietic fibroblasts (HEK293s) infected with the indicated recombinant DENV-2 strains.

To determine whether DENV-2 transcripts could be targeted by the miRNA machinery, we first attempted to see whether the presence of miR-142 could prevent virus production from the *in vitro* transcription (IVT) of the 142t cDNA clone. BHKs, expressing vector or p142, were electroporated with IVT products from wt, ctrl, or 142t DENV-2 cDNA clones. Twenty-four hrs post transfection (hpt), miR-142 production was evident by northern blot, albeit lower than observed in bone-marrow derived primary macrophages (BMMs) (Figure 3A). Given the production of miR-142, we evaluated NS5 protein levels from the IVT product to determine the degree of transcript targeting (Figure 3B). Consistent with previous work demonstrating miRNA-mediated virus attenuation [31], NS5 expression derived only from 142t IVT product was abrogated in a miRNA-specific manner (Figure 3B). This result would suggest that virus synthesis from the 142t IVT product was blocked in the presence of miR-142.

Next, we sought to determine whether miR-142 could attenuate 142t in the context of infection. To this end, fibroblasts expressing vector or p142 were infected with wt, ctrl, or 142t strains, and assessed for virus products. As evident by NS5 expression, miR-142 abolished replication of the 142t strain, while exuding no impact on wt or ctrl strains (Figure 3C). It is noteworthy that residual levels of NS5 for the 142t strain likely reflect replication in cells not successfully transfected with p142, a constraint that would not be in place during an endogenous infection of a hematopoietic cell. Taken together, these data clearly demonstrate that insertion of miRNA target sites into the 3′UTR of DENV-2 renders the virus susceptible to miRNA expression.

In an attempt to ascertain the mechanism underlying miR-142-mediated attenuation, we modified the miR-142 target sites to render the DENV genome resistant to canonical miRNA binding and Ago2 cleavage. To this end, the nucleotides complementary to positions 3 and 10 of miR-142 were mismatched in the virus targets to destroy the seed sequences and cleavage sites, respectively [47]. To assess whether this mutated 142t strain, herein referred to as 142tm, was targeted by miR-142, we compared NS5 synthesis in the presence and absence of the target miRNA (Figure 3D). Surprisingly, virus production from the 142tm strain was still abrogated in a miR-142-specific manner, despite harboring two critical mismatches in the miRNA targeting sites. As loss of NS5 in the 142tm strain maintained miR-142-specificity, these results would suggest that RISC was still engaging on the mutated targets in a non-canonical manner, perhaps as a result of the remaining extensive complementarity. To determine the underlying mechanism responsible for attenuation of the 142tm strain, we examined the levels of genomic viral RNA to discern between RNA degradation and translational inhibition. Quantitative RT-PCR analysis demonstrated that, at the level of RNA, the amount of miR-142-mediated repression of the 142tm virus was significantly (p = 0.0178) lower than that of 142t, suggesting the mode of attenuation may no longer be RNA cleavage, but may involve a form of translational repression (Figure 3E). This translational repression could be the result of extensive 3′ miRNA complementarity acting in a canonical manner or could be the result of miR-142/RISC sterically blocking the association of the 5′ and 3′ ends, preventing cyclization and amplification of the virus genome. While future studies will be required to ascertain the exact mechanism of 142tm attenuation, these data strongly demonstrate that inhibition of virus replication occurs in a miR-142-dependent manner. As the 142tm virus showed decreased silencing activity, subsequent characterization focused only on the comparison between ctrl and 142t strains.

As cells of hematopoietic lineage are the natural sites of DENV infection and express high levels of miR-142 (Figure 3A) [1], [44], we aimed to determine whether we could observe differential cell-specific attenuation between the ctrl and 142t strains. To this end, we compared infections of the recombinant DENV-2 strains in two hematopoietic lineages, B cells and BMMs, as well as a non-hematopoietic fibroblast lineage, HEK293s (Figure 3F). Consistent with exogenous targeting by miR-142, infection of hematopoietic cell types demonstrated selective attenuation of the 142t strain, while infection of the non-hematopoietic cells demonstrated no change in NS5 levels between ctrl- and 142t-virus infected samples (Figure 3F). Taken together, these data demonstrate that the incorporation of miR-142 target sites into the 3′UTR of DENV-2 confers endogenous attenuation of the virus in a cell-specific manner.

**Figure 4:**
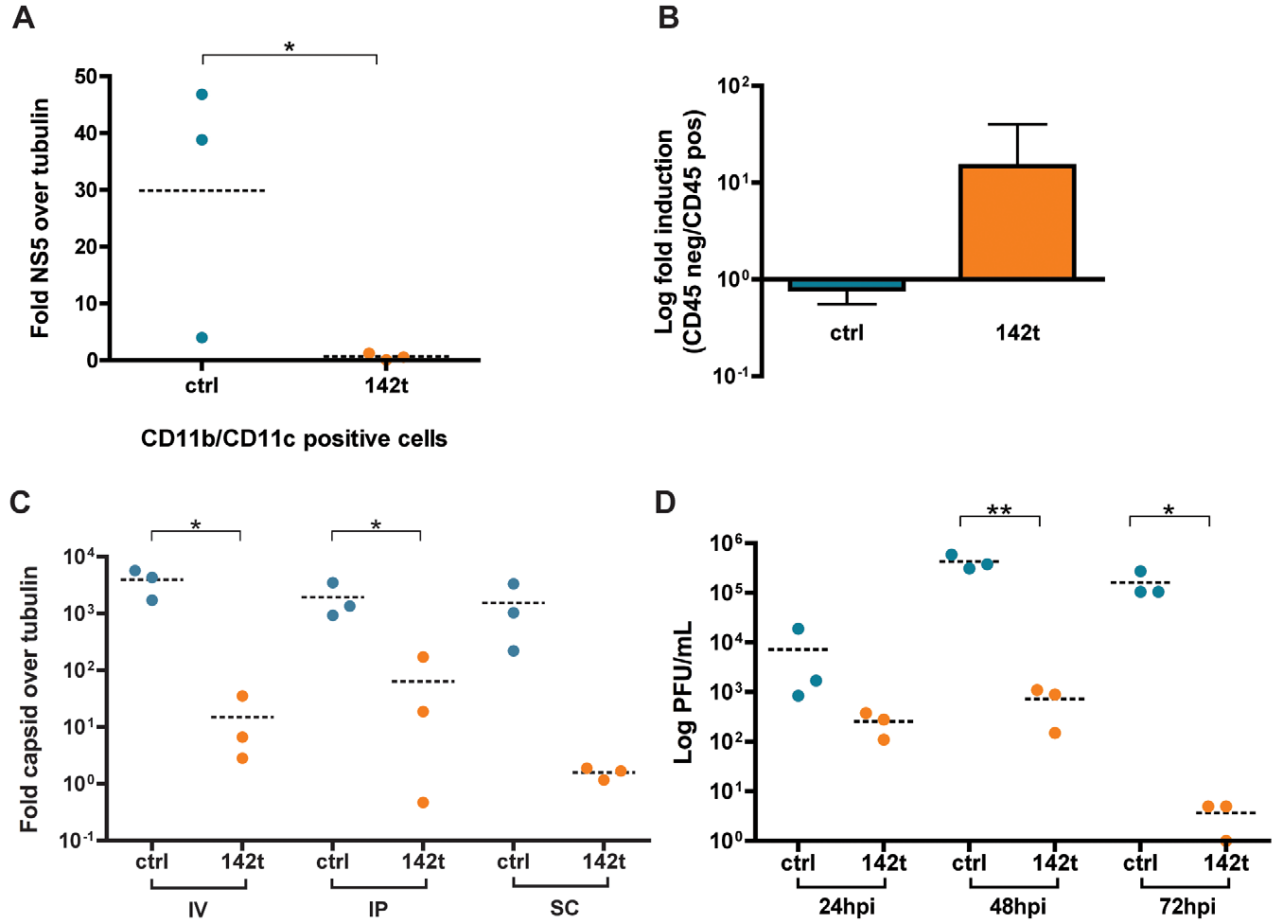
*In vivo* knockdown of miR-142-targeted DENV-2. (A) Quantitative RT-PCR (qRT-PCR) on NS5 and tubulin from CD11b^+^ and CD11c^+^ cells from splenocytes of *Ifnar1^−/−^/Il28r^−/−^* mice inoculated with either ctrl or 142t strains via intravenous (IV) injection. (B) qRT-PCR for DENV capsid in CD45^−^ versus CD45^+^ splenocytes represented as a ratio for ctrl and 142t virus infections in *Ifnar1^−/−^/Il28r^−/−^* mice. (C) qRT-PCR on spleens from *Ifnar1^−/−^/Il28r^−/−^* mice infected intraveneously (IV), intraperitonealy (IP), and subcutaneously (SC) for 48 hrs. (D) Viral titers from spleens of *Ifnar1^−/−^/Il28r^−/−^* mice infected as in (A) for 24, 48, and 72 hpi. (Statistical significance: *, P<0.05, **, P<0.01) Each data point represents an individual animal.

As human DENV isolates currently lack an adequate animal model that recapitulates human pathogenesis during infection, we chose *Ifnar1^−/−^/Il28r^−/−^* mice to investigate our recombinant viruses *in vivo*. These mice lack the ability to respond to type I and III interferon and are, thus, more susceptible to virus infection [48], [49]. To ensure that the *in vitro* and *ex vivo* data reflected *in vivo* attenuation, we first infected mice with ctrl and 142t viruses and isolated CD11b^+^ and CD11c^+^ macrophages and DCs, respectively (Figure 4A). qRT-PCR analysis of RNA derived from these cells demonstrated minimal detection of NS5 transcripts derived from the 142t strain and a significant decrease compared to ctrl virus. These data support our *in vitro* data, where 142t virus replication is excluded in hematopoietic cell types.

To examine *in vivo* replication of the 142t virus in non-hematopoietic compartments, we sorted splenocytes from infected mice for CD45-expressing cells, and assessed viral gene expression in each population (Figure S4A). The nature of this experiment however is complicated by the fact that, should hematopoietic cells be required for virus dissemination, we would anticipate lower virus titers in non-hematopoietic fractions as well. As anticipated, 142t virus growth was attenuated in the CD45^+^ hematopoietic fraction. In addition, the CD45^−^, non-hematopoietic fraction demonstrated a decrease in 142t virus growth as compared to ctrl, supporting that dissemination of the virus is dependent on its ability to replicate in hematopoietic cell types. To account for this lack of dissemination, we compared the relative level of virus growth between hematopoietic and non-hematopoietic populations, and determined that the 142t virus displayed enhanced replication in CD45^−^, non-hematopoietic cells (Figure 4B). Taken together, these data suggest that the 142t virus is not attenuated in non-hematopoietic cells, but that titers are impaired as a result of decreased hematopoietic replication.

Following verification of *in vivo* targeting, we further assessed dissemination of the ctrl and 142t strains in *Ifnar1^−/−^/Il28r^−/−^* mice. To this end, three different routes of inoculation were administered to assess virus replication in both liver and spleen. Different cohorts of mice were administered virus by either intraperitoneal (IP), intraveneous (IV), or subcutaneous (SC) injection (Figure 4C, Figure S4B). For IP administration, qRT-PCR analysis of the spleen revealed an ∼1.5-log reduction in viral transcript levels of the 142t strain as compared to those given ctrl virus (Figure 4C). This attenuation was further enhanced by IV and SC injection, routes that better simulate natural inoculation. Interestingly, decreased amounts of NS5 transcript was also evident in the liver (Figure S4B), where miR-142 expression is reduced compared to the spleen [50]. Taken together, these *in vivo* results suggest that virus levels in the liver reside predominantly in resident macrophages and DCs, and that perhaps these cells are needed to maintain basal levels of virus replication in non-hematopoietic cells. To further validate the reduction of viral growth *in vivo*, viral titers from infected mice corroborated qRT-PCR data by demonstrating an approximate three-log reduction in titers from spleen and liver (Figure 4D, Figure S4C). Viral titers were undetectable in heart, lung, and brain from these mice, demonstrating that the spleen and liver were the primary sites of virus replication in the context of this animal model.

**Figure 5:**
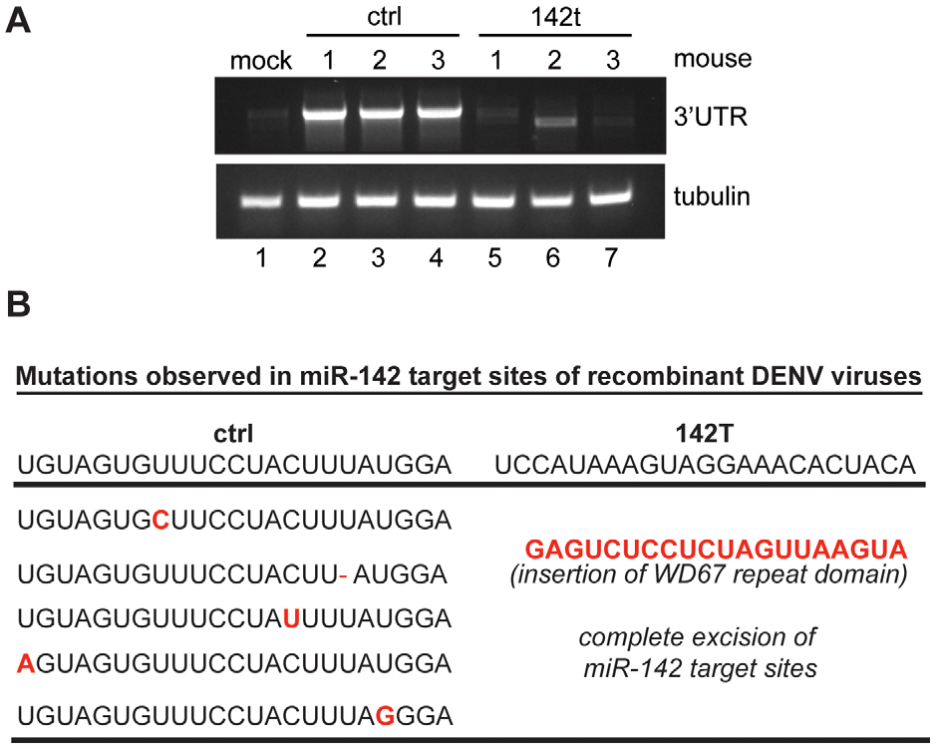
*In vivo* escape mutants of miR-142-targeted DENV-2. (A) RT-PCR on spleen samples from *Ifnar1^−/−^/Il28r^−/−^* mice infected with either ctrl or 142t viruses. (B) Mutations identified in *Ifnar1^−/−^/Il28r^−/−^* mice infected with ctrl and 142t viruses. Depicted mutations map to miR-142 complementary sites in the targeted and untargeted orientations. Notations in red designate nucleotide changes.

As low levels of 142t virus were evident in both the spleen and the liver, we next sought to determine whether this reflected a non-hematopoietic reservoir for the virus or whether this was evidence of virus escape. In an effort to characterize the genotype of the recombinant DENV viruses over the course of infection, we amplified the 3′UTR of the ctrl and 142t viruses derived from *in vitro* and *in vivo* infections (Figure S5 and Figure 5A). To this end, non-hematopoietic fibroblasts expressing varying levels of miR-142 were infected for 3′UTR sequence analysis. *In vitro*, 142t-specific transcripts were only detectable in conditions where miR-142 expression was decreased, a result that could reflect lower transfection efficiency or insufficient miRNA levels. To assess the 3′UTR sequence *in vivo*, splenocytes from infected mice were analyzed, demonstrating abundant transcript levels of the ctrl virus and significantly lower levels of the 142t virus (Figure 5A). Furthermore, the only product derived from the 142t virus migrated faster during gel electrophoresis, suggesting it had been truncated. Sequence analyses of transcripts from these two infections demonstrated an unbiased mutation frequency *in vitro* that was comparable between the two viral cohorts, suggesting the mutations were a reflection of low polymerase fidelity, rather than a result of evolutionary constraint. In contrast, sequence analyses of the transcripts found *in vivo* demonstrated a complete absence of 142t virus. Rather, virus species remaining displayed loss of all four miRNA target sites either by complete excision or by replacement with a small host RNA fragment (Figure 5B). The sequence analysis performed here explains why low levels of 142t virus was detected by qRT-PCR in Figure 4, as this assay measured levels of NS5, which encompasses any escape mutants present during infection. Our data in Figure 5 reveals that the percentage of 142t virus represented is, in fact, much lower. Furthermore, this is unlikely attributable to the presence of quasi-species in our virus stocks, as these mutations were not observed *in vitro* (Figure S5B).

Previous efforts aimed at defining the tropism of DENV infection have focused on the detection of virus components in various tissues of the host. Unfortunately, distinguishing between active DENV replication within a cell versus the presence of virus due to cellular engulfment has been difficult. Here we take an innovative approach to addressing this question with the generation of a virus that is selectively attenuated in a cell-type specific manner. Through the exploitation of hematopoietic-specific miR-142, we were able to exclude the replication of DENV at its major sites of replication *in vivo*, and demonstrate that these cell populations are critical for dissemination of the virus to other tissues. Our initial *in vitro* work clearly demonstrated the specificity of the system and provided a foundation for the subsequent studies performed in mice. The technology enabled a precise mechanism to eliminate primary target cells of DENV replication and study the effects in the context of a dynamic *in vivo* infection.

An additional noteworthy finding that resulted from this study centers on the mechanism of miRNA-mediated attenuation. While attenuation of the 142t strain is presumably the result of target RNA cleavage by Ago2, the repression of 142tm is an enigma. The data supports that 142tm attenuation occurs less at the level of RNA than it does at the level of protein, making the underlying molecular biology responsible for this repression unclear. While the extensive complementarity may be responsible for some level of post-transcriptional silencing, the complete loss of the seed sequence makes this unlikely. An alternative hypothesis would be that the level of complementarity is sufficient to recruit miR-142-containing RISC, but not sufficient to confer post- transcriptional silencing. In this model, RISC-binding may do nothing more but provide a steric hinderance to the virus. To undergo replication, DENV, as well as other flavivirus genomes, cyclize through complementarity at conserved elements in the 5′ and 3′ ends of the viral RNA [51]. As such, RISC function may be at the level of physically disrupting proper folding of the virus genome. This latter model is supported by the isolation of escape mutants. Rather than identifying single point mutations in the seed or center sites of each target, the only escape mutants isolated were viruses in which the complete targeting cassette was excised. Taken together, this suggests that miRNA-mediated attenuation of viruses may be occurring both through transcriptional silencing, and through the steric interference of RNA folding. Future work to address this possibility is ongoing.

In closing, this technology has become a unique and effective tool to study cell populations involved in the DENV life cycle, and can be easily applied to other viruses to examine the relevance of cellular subsets involved in virus replication. In this regard, it is interesting that restricting hematopoietic replication of DENV-2 prevents overall dissemination of the virus suggesting that non-hematopoietic primary cells may not be productively infected *in vivo* or that macrophages and DCs are essential for viral spread. Altogether, the successful attenuation of DENV in a cell-specific manner suggests this technology may be exploited for studying the relative contributions of cell subsets to virus pathogenesis *in vivo*.

## Materials and Methods

### Ethics statement

All animal studies were approved by the Animal Care and Use Committee of Mount Sinai School of Medicine and were performed in compliance with relevant institutional policies, the Association for the Accreditation of Laboratory Animal Care guidelines, the National Institutes of Health regulations, and local, state, and federal laws.

### Plasmid construction and virus rescue

A full-length infectious cDNA clone of DENV-2 (16681), pD2/IC-30P-A, was used as a template for site-directed mutagenesis (SDM) to introduce a unique *AflII* restriction enzyme site for subsequent insertion of miR-142 target sites into the 3′ NCR [52]. The pD2/IC-30P-A construct was a kind gift from Richard Kinney (Arbovirus Disease Branch, CDC, Fort Collins, CO). The *AflII* site was generated with complementary SDM primers resulting in the following sequence: 5′-TAGAAAGCTTAAGTAACATGAAA-3′ (*AflII* site is underlined). Target sites for miR-142 have been described elsewhere [53] and were cloned into the *AflII* sites in both the forward (142t DENV-2) and reverse (ctrl DENV-2) orientations. For the 142m strain, the following complementary oligonucleotides were annealed and cloned into the AflII site: Forward 5′-GGCTTAAGTCCATAAAGTAGGCAACACTCCAAGGCGATCCATAAAGTAGGCAACACTCCAGCGGCCGCTCC-3′, Reverse 5′- CCCTTAAGTGGAGTGTTGCCTACTTTATGGAGAGCCCTGGAGTGTTGCCTACTTTATGGAGCGGCCGCTGG -3′. Recombinant pD2/IC-30P-A clones were linearized with *XbaI*, *in vitro* transcribed with T7, and electroporated into BHK cells as previously described [52]. Viral titers were determined by 1% agarose-based plaque assays performed in Vero cells.

### Fluorescence-activated cell sorting

Transfected and infected HEK293s were harvested 48 hpi and analyzed for GFP fluorescence on the Becton Dickinson FACS Caliber and mean fluorescence intensity was calculated using the Flowjo analysis software. Experiments were performed in triplicate and mean fluorescence intensity for GFP was calculated as a percentage of the vector control.

### Cell culture, infections, and plaque assays

Unless otherwise specified, all mammalian cell lines were cultured in DMEM/FBS. C6/36 cells were cultured in RPMI 1640/FBS and maintained at 33°C. BHK and C6/36 cells were infected with either wt, ctrl, or 142t DENV-2 at an MOI of 1 in serum-free DMEM. The inoculum was allowed to adsorb for two hrs at 37°C, washed and replaced with DMEM (5% FBS). Infection for plaque assays were performed as described above in BHKs in duplicate with 1∶10 serial dilutions of virus stocks. Infected mouse organs were harvested and homogenized in 1× PBS, and frozen at −80°C prior to plaque assay. After 2 hrs of adsorption, an overlay of 1% agarose/DMEM was added to the cells and allowed to incubate for 6 days at 33°C. Cells were stained using 1% crystal violet.

### Transfection and electroporation

Cells were transfected in suspension using Lipofectamine 2000 for HEK293s (Invitrogen) and Lipofectamine LTX (Invitrogen) for BHKs as per manufacturer's instructions. For studies involving knockdown of DENV IVT products, BHKs were transfected with vector or p142 for 24 hrs, followed by electroporation of DENV-2 IVT products for 12 (Figure 3E), 24 or 48 hrs with the Amaxa nucleofector (Lonza) as per manufacturer's instructions. For virus rescue, 5×10^6^ BHKs were electroporated with DENV-2 IVT products and seeded into 10 cm plates. Six days post electroporation, supernatant was harvested and stored at −80°C.

### *In vivo* infections

*Ifnar1^−/−^/Il28r^−/−^* mice have been described elsewhere [48]. Mice were given 2×10^6^ PFU for IP, 4×10^5^ PFU for IV, and 2×10^5^ PFU for SC injections. Indicated organs were removed from animals 48 hpi. All experiments involving animals were performed in accordance with the Mount Sinai School of Medicine Institutional Animal Care and Use Committee.

### Magnetic-activated cell sorting

Spleens from infected *Ifnar1^−/−^/Il28r^−/−^* mice were processed into single cell suspensions and stained with anti- CD11b (Miltenyi Biotech), CD11c (Miltenyi Biotech), or CD45 microbeads (Miltenyi Biotech). Positive and negative cell fractions were isolated using autoMACS Pro Separator (Miltenyi Biotech) followed by RNA isolation.

### Western blot and qRT-PCR analysis

Protein and RNA cellular extract were harvested as previously described (Perez et al, 2009). NS5 antibody (anti-NS5, rabbit polyclonal, a kind gift from A. Garcia-Sastre) and actin (anti- actin, Abcam,) were used at a concentration of 1 microgram per milliliter in 5% milk. Secondary rabbit or mouse antibodies (GE Healthcare) were used at 1∶5000 dilutions for 1 hr at room temperature. Immunobilon Western Chemiluminescent HRP Substrate (Millipore) was used as per manufacturers instructions. Quantitative RT-PCR (qRT-PCR) analysis was performed with random hexamers using Superscript II (Invitrogen) and cDNA samples was performed using KAPA SYBR FAST qPRC Master Mix (KAPA Biosystems). PCR reactions were performed on a Mastercycler ep realplex (Eppendorf). α-Tubulin and actin primers were used as endogenous housekeeping genes for mammalian and mosquito cultures, respectively. Delta delta cycle threshold (ΔΔCT) values were calculated with replicates over α-tubulin or actin. Values represent the fold change over mock-infected samples. Sequences for PCR primers used: NS5: 5′- ACAAGTCGAACAACCTGGTCCAT-3′, 5′-GCCGCACCATTGGTCTTCTC-3′, α-tubulin: 5′-TGCCTTTGTGCACTGGTATG-3′, 5′- CTGGAGCAGTTTGACGACAC-3′, actin: 5′-GCACTGGACTTTGAACAGGAAATG-3′, 5′-AGGAACGATGGCTGGAAGAGAG-3′, capsid: 5′-TGGTGGCGTTCCTTCGTTTC-3′, 5′ GCATCCTTCCAATCTCTTTCCTG-′3, and DENV 3′UTR: 5′- TCCCTTATAGGCAATGAAGAATACA-3′, 5′-TTATGATGGCCTGACTTCTTTTAAC-3′.

### Sequencing of escape mutants

cDNA from BHKs transfected with 1.5 micrograms of p142 and infected with either ctrl or 142t viruses for 24 hrs was reverse transcribed with random hexamers using Superscript II (Invitrogen). Virus transcript was PCR-amplified with Econotaq PLUS green (Lucigen) using primers specific for the 3′UTR of DENV: forward 5′-TTTGGGGAAGTCTTACGC-3′, reverse 5′-GTTGCTGCGATTTGTAAGG-3′. Hamster actin primers were included as a loading control: forward 5′-TCTACAACGAGCTGCG-3′, reverse 5′-CAATTTCCCTCTCGGC-3′. Twenty PCR cycles were performed with the following conditions: 94°C for 10 sec, 58°C for 30 sec, 72 for 1.5 min. Bands were gel purified using the Qiagen gel extraction kit and cloned into pCR-TOPO 2.1 (Invitrogen) following manufacturer's protocols. Fifteen random clones were submitted for sequencing with the M13R primer. Sequences were then aligned to miR-142 complementary sites in the targeted and untargeted orientation. Mutations identified in these regions were noted. For escape mutants found *in vivo*, reverse transcription was performed on cDNA from spleens of *Ifnar1^−/−^/Il28r^−/−^* mice infected with ctrl or 142t viruses for 48 hrs. PCR was performed as described above for 40 cycles. Bands were gel purified and cloned as described above.

### Small RNA northern blot analysis

Small RNA northern blotting and probing were performed on total RNA samples as described previously (Perez et al, 2009). Probes include: miR-142: 5′- TCCATAAAGTAGGAAACACTACA-3′, U6: 5′-GCCATGCTAATCTTCTCTGTATC -3′.

### Statistical analysis

Statistical significance was calculated using a two-tailed, unpaired T test. Data considered significant demonstrated p values less than 0.05.

## Supporting Information

Figure S1 **DENV-2 does not disrupt endogenous or exogenous miRNA production.** (A) Northern blot on HEK 293s transfected with p124 and infected with the indicated recombinant DENV-2 viruses at an MOI of 1 48 hpi. Blot was probed for miR-124 expression (B). Northern blot on HEK293s and BHKs infected as in (A). Blot was probed for endogenous levels of miR-93.
(TIF)

Figure S2 **Cloning strategy for incorporation of target sites into the DENV2 3′UTR.** Site-directed mutagenesis was performed to generate an *AflII* restriction enzyme site 7 nucleotides (nt) downstream of the NS5 open reading frame. A 157 nt cassette containing four miR-142 target sites in tandem were cloned into the *AflII* site to generate a miR-142-targeted DENV-2 virus (142t). Viruses containing the same cassette in reverse and no insertions were also generated to serve as controls (ctrl and wt viruses, respectively).
(TIF)

Figure S3 **Recombinant DENV2 viruses grow to similar titers in mosquito cells.** Multicycle replication kinetics was performed in C6/36 cells for the indicated time points.
(TIF)

Figure S4 ***In vivo*** **knockdown of miR-142-targeted DENV-2.** (A) Quantitative RT-PCR (qRT-PCR) on Capsid and tubulin for CD45^+^ and CD45^−^ cells from splenocytes of *Ifnar1^−/−^/Il28r^−/−^* mice inoculated with either ctrl or 142t strains via intravenous (IV) injection for 24 hrs. (B) Viral titers from livers of *Ifnar1^−/−^/Il28r^−/−^* mice inoculated with either ctrl or 142t strains via IV injection for 24, 48, and 72 hpi. Each dot represents one animal.
(TIF)

Figure S5 ***In vitro*** **escape mutants of miR-142-targeted DENV-2.** (A) RT-PCR on RNA derived from BHKs transfected with varying amounts of p142, and infected with either ctrl or 142t viruses. (B) Mutations identified in p142-transfected BHKs infected with ctrl and 142t viruses. Depicted mutations map to miR-142 complementary sites in the targeted and untargeted orientations. Notations in red designate nucleotide changes.
(TIF)
