# Supplementary figures and images for: Replication in Cells of Hematopoietic Origin Is Necessary for Dengue Virus Dissemination
Source: PLoS Pathog. 2012 Jan 5;8(1):e1002465. doi: 10.1371/journal.ppat.1002465 (PMC3252368; doi:10.1371/journal.ppat.1002465)

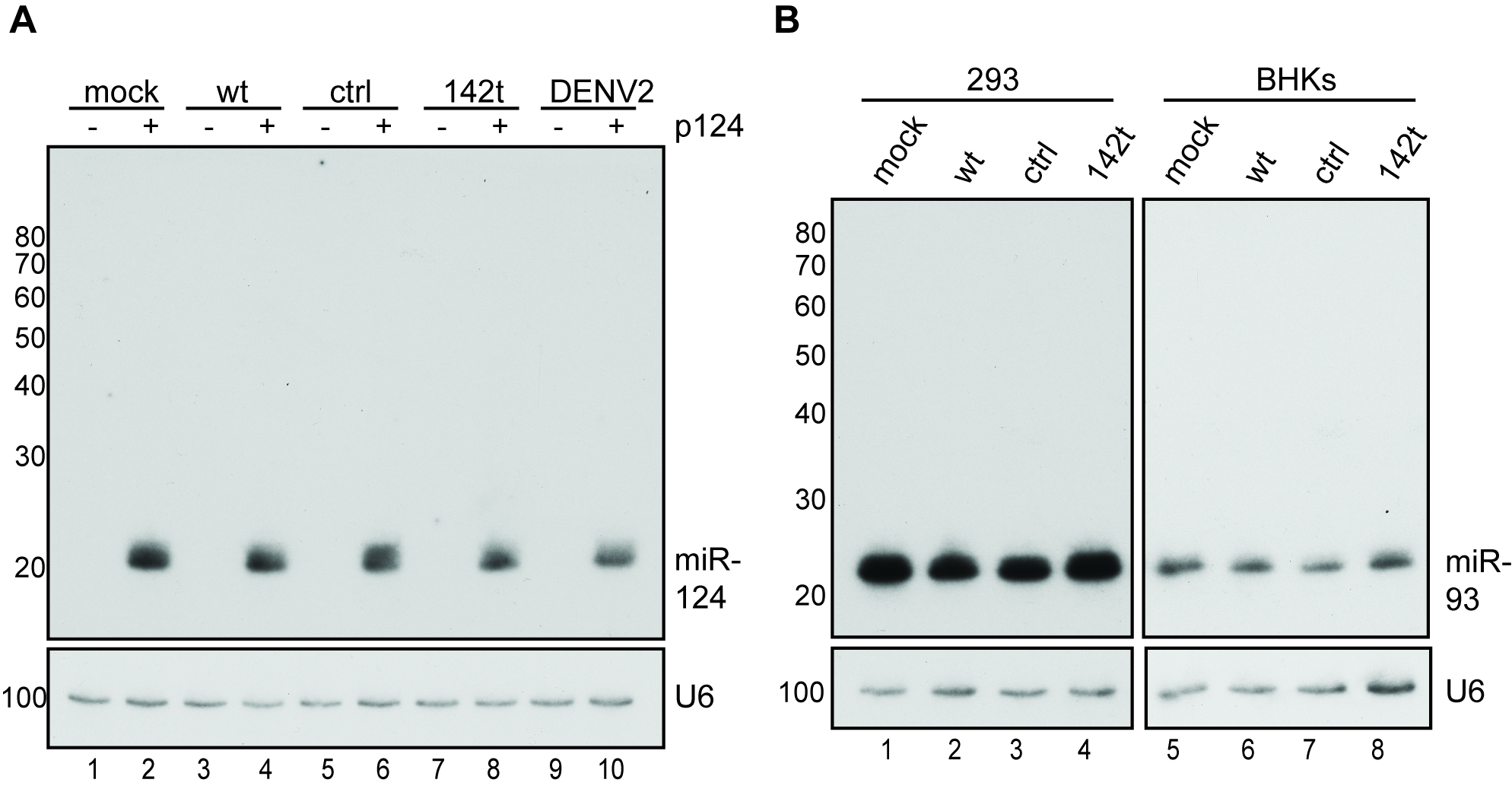

Supplement: Figure S1 — DENV-2 does not disrupt endogenous or exogenous miRNA production. (A) Northern blot on HEK 293s transfected with p124 and infected with the indicated recombinant DENV-2 viruses at an MOI of 1 48 hpi. Blot was probed for miR-124 expression (B). Northern blot on HEK293s and BHKs infected as in (A). Blot was probed for endogenous levels of miR-93. (TIF) [file ppat.1002465.s001.tif]

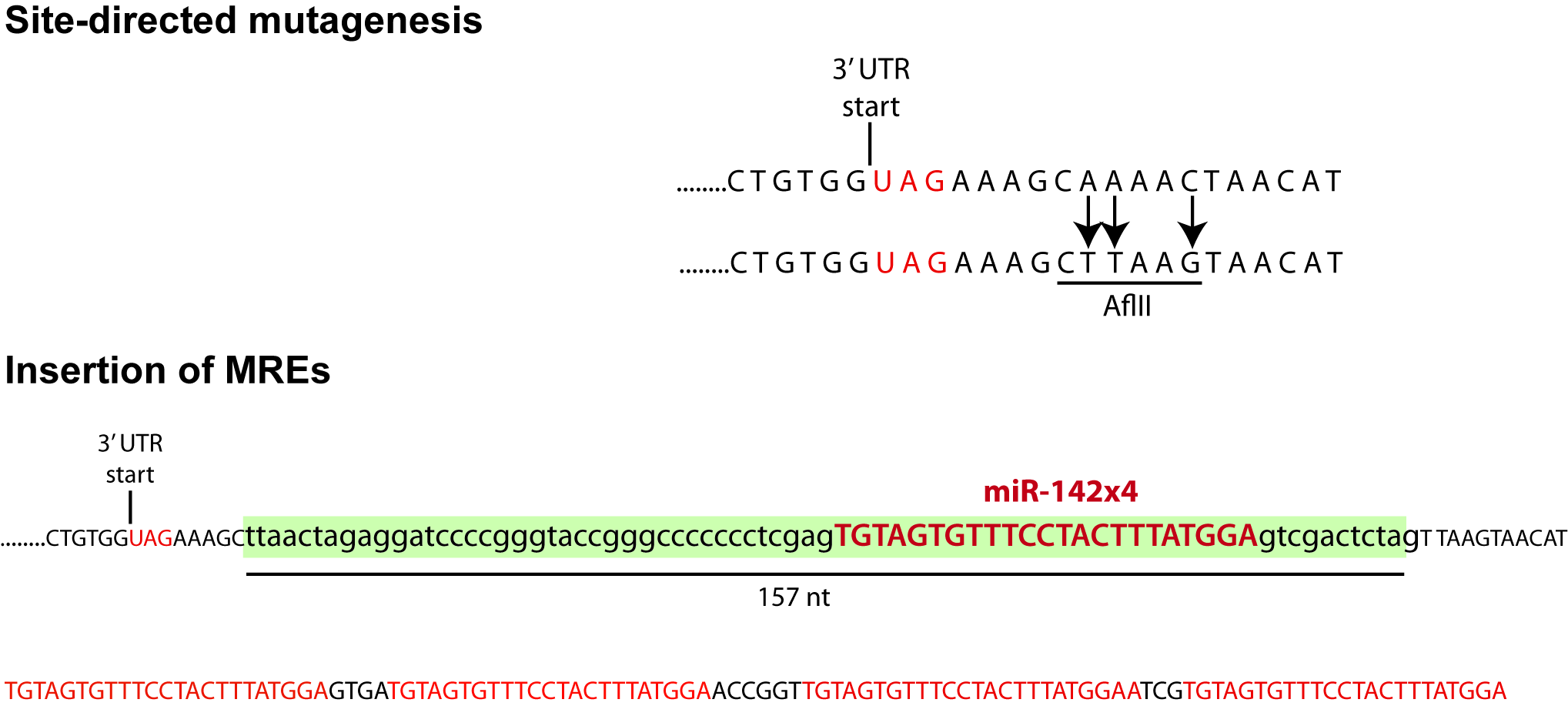

Supplement: Figure S2 — Cloning strategy for incorporation of target sites into the DENV2 3′UTR. Site-directed mutagenesis was performed to generate an AflII restriction enzyme site 7 nucleotides (nt) downstream of the NS5 open reading frame. A 157 nt cassette containing four miR-142 target sites in tandem were cloned into the AflII site to generate a miR-142-targeted DENV-2 virus (142t). Viruses containing the same cassette in reverse and no insertions were also generated to serve as controls (ctrl and wt viruses, respectively). (TIF) [file ppat.1002465.s002.tif]

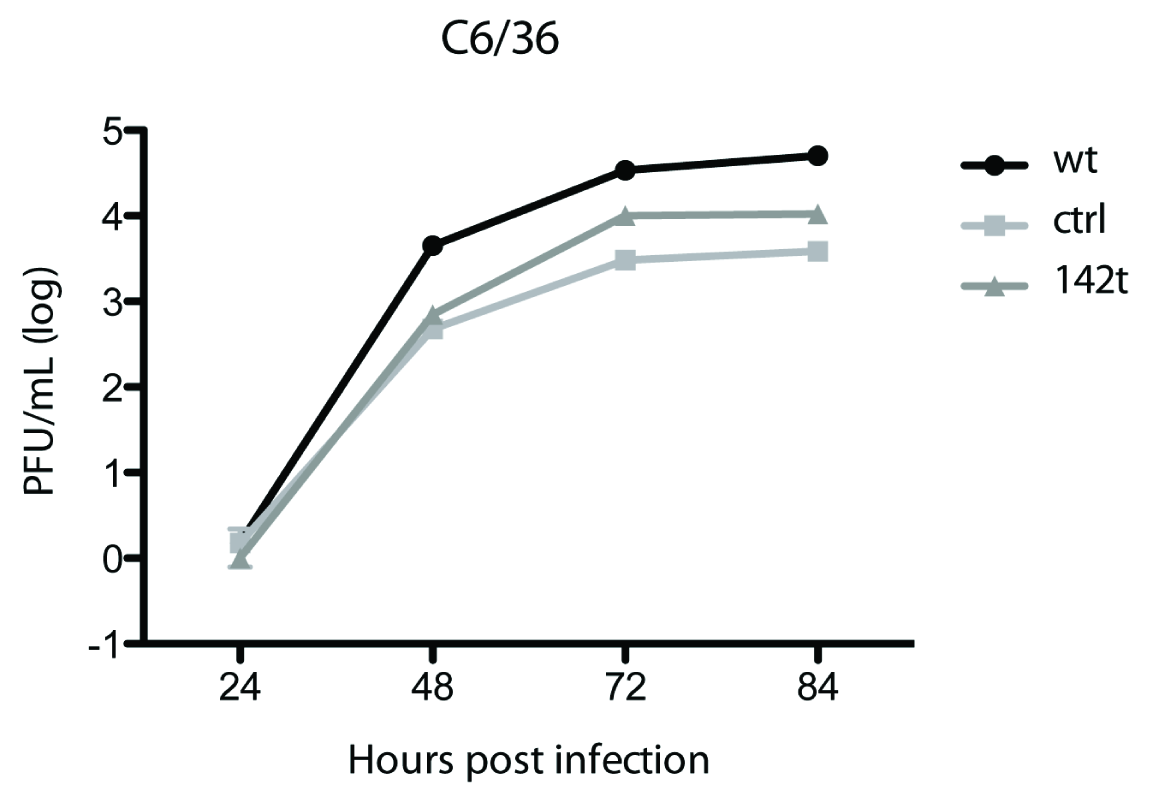

Supplement: Figure S3 — Recombinant DENV2 viruses grow to similar titers in mosquito cells. Multicycle replication kinetics was performed in C6/36 cells for the indicated time points. (TIF) [file ppat.1002465.s003.tif]

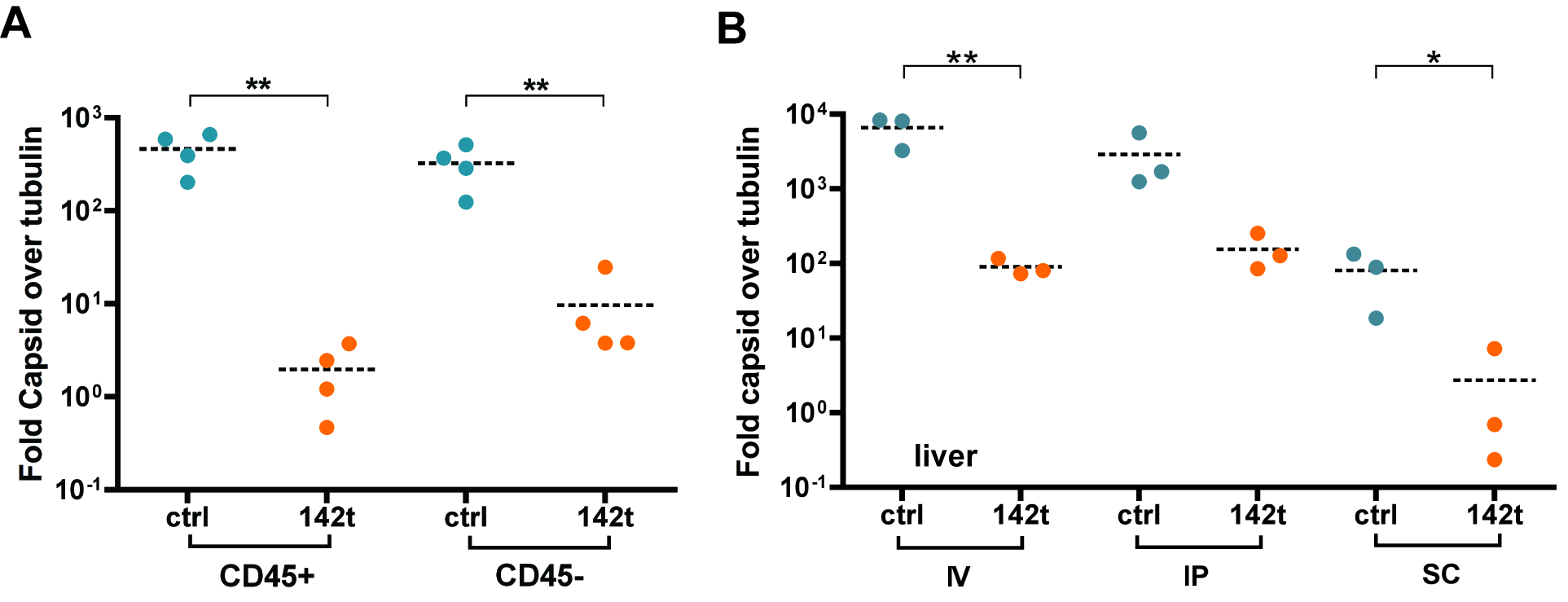

Supplement: Figure S4 — In vivo knockdown of miR-142-targeted DENV-2. (A) Quantitative RT-PCR (qRT-PCR) on Capsid and tubulin for CD45+ and CD45− cells from splenocytes of Ifnar1−/−/Il28r−/− mice inoculated with either ctrl or 142t strains via intravenous (IV) injection for 24 hrs. (B) Viral titers from livers of Ifnar1−/−/Il28r−/− mice inoculated with either ctrl or 142t strains via IV injection for 24, 48, and 72 hpi. Each dot represents one animal. (TIF) [file ppat.1002465.s004.tif]

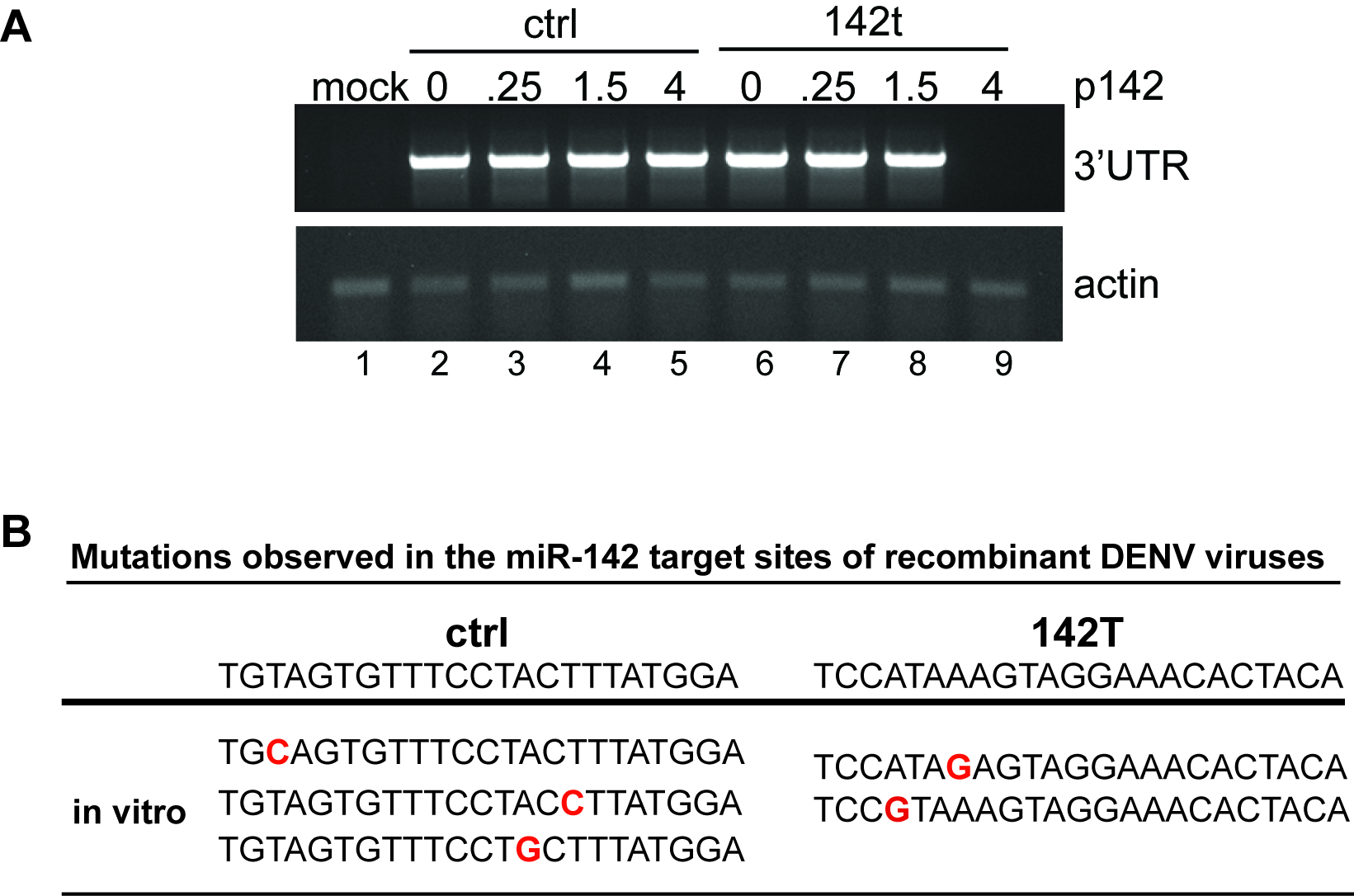

Supplement: Figure S5 — In vitro escape mutants of miR-142-targeted DENV-2. (A) RT-PCR on RNA derived from BHKs transfected with varying amounts of p142, and infected with either ctrl or 142t viruses. (B) Mutations identified in p142-transfected BHKs infected with ctrl and 142t viruses. Depicted mutations map to miR-142 complementary sites in the targeted and untargeted orientations. Notations in red designate nucleotide changes. (TIF) [file ppat.1002465.s005.tif]
